# Mine, Mine, Mine: Self-Reference and Children’s Retention of Novel Words

**DOI:** 10.3389/fpsyg.2018.00958

**Published:** 2018-06-12

**Authors:** Emma L. Axelsson, Rachelle L. Dawson, Sharon Y. Yim, Tashfia Quddus

**Affiliations:** ^1^Research School of Psychology, ANU College of Health & Medicine, Australian National University, Canberra, ACT, Australia; ^2^Department of Psychology, Uppsala University, Uppsala, Sweden

**Keywords:** referent selection, self-reference effect, word learning, eye-tracking, toddlers

## Abstract

Adults demonstrate enhanced memory for words encoded as belonging to themselves compared to those belonging to another. Known as the self-reference effect, there is evidence for the effect in children as young as three. Toddlers are efficient in linking novel words to novel objects, but have difficulties retaining multiple word-object associations. The aim here was to investigate the self-reference ownership paradigm on 3-year-old children’s retention of novel words. Following exposure to each of four novel word-object pairings, children were told that objects either belonged to them or another character. Children demonstrated significantly higher immediate retention of self-referenced compared to other-referenced items. Retention was also tested 4 h later and the following morning. Retention for self- and other-referenced words was significantly higher than chance at both delayed time points, but the difference between the self- and other-referenced words was no longer significant. The findings suggest that when it comes to toddlers’ retention of multiple novel words there is an initial memory enhancing effect for self- compared to other-referenced items, but the difference diminishes over time. Children’s looking times during the self-reference presentations were positively associated with retention of self-referenced words 4 h later. Looking times during the other-reference presentations were positively associated with proportional looking at other-referenced items during immediate retention testing. The findings have implications for children’s memory for novel words and future studies could test children’s explicit memories for the ownership manipulation itself and whether the effect is superior to other forms of memory supports such as ostensive naming.

## Introduction

Children are regularly exposed to novel words and objects, and the apparent speed at which toddlers acquire words belies the complex and remarkable process of encoding and retaining novel words. Word learning involves multiple tasks such as segmenting words from within speech streams (Horst and Samuelson, 2008), matching word-forms with their respective objects, and retaining novel word-object associations for retrieval in future encounters (Munro et al., 2012). Children are not always explicitly told the labels for objects, but when presented with a novel object in the context of familiar objects, children tend to link a novel word with a novel object (Carey, 1978). One term for this is fast mapping and despite children’s speed and accuracy at fast mapping (Mervis and Bertrand, 1994; Halberda, 2003), they demonstrate difficulty in remembering novel word-object associations particularly when there are multiple novel words (Axelsson and Horst, 2013). In a study by Horst and Samuelson (2008), 2-year-old children were highly accurate at fast mapping eight novel objects, but after a 5-min delay children’s retention was at chance. When Horst and Samuelson ostensively labeled the novel objects after each fast mapping trial, children demonstrated above-chance retention for four of the objects. Other types of memory supports can also enhance children’s retention of recently fast-mapped words such as enhancing attention to the target when it is renamed (Axelsson et al., 2012), making the target object more salient (Vlach and Sandhofer, 2012), or repeating the target word several times (Gurteen et al., 2011).

In studies of adults’ memory, Rogers et al. (1977) found that adults recalled more words they had evaluated as describing themselves as compared to words they evaluated semantically (synonyms), phonemically (rhyming), or structurally (capitals versus lower case). Referred to as the self-reference effect (SRE), recall for self-referenced words also tends to be better than for those coded in relation to another person, but this effect is smaller than the comparison between self-referenced items and semantically encoded items (see Symons and Johnson, 1997 for a review). Cunningham et al. (2008) also found better recognition of items presented as ‘belonging to themselves’ than those belonging to another character. There are also higher levels of specific as opposed to ambiguous memory for self-referenced items compared to other-referenced, suggesting that there is an episodic aspect to memory for self-referenced items (Conway et al., 2001; van den Bos et al., 2010).

Klein and Loftus (1988) argued that because knowledge of oneself is vast and rich in detail, self-referential information is more readily elaborated and organized during encoding. Greater *elaboration* suggests that the semantic properties of information is encoded in greater detail and better *organization* suggests that information is better linked to existing categories of knowledge; and both elaboration and organization during encoding creates multiple pathways to retrieval (Klein, 2012). van den Bos et al. (2010) further argued that ownership might be associated with increased attention and emotional arousal to self-referenced items as well as greater potential for considering the details of the items in relation to oneself.

There is strong evidence of the SRE in adults (Symons and Johnson, 1997; Klein, 2012), but there are fewer studies with children and the age at which an SRE emerges is unclear. Cunningham et al. (2013) asked pairs of 4- to 6-year-old children to sort images into boxes that were labeled as theirs or belonging to a partner. Children were asked to imagine that they owned the specific images that went into their box. In a subsequent recognition task, children demonstrated superior memory for the items that were placed in their box compared to items that went into their partner’s box. Similarly, Sui and Zhu (2005) found that 5- and 10-year-olds, but not 4-year-olds, remembered items classified as their own better than items classified as someone else’s; while Ross et al. (2011) found that children as young as three demonstrated a memory bias for self-owned items.

The current study was aimed at investigating whether the SRE can be applied to children’s retention of *novel* words. A fast mapping paradigm was employed to expose children to the novel words. Fast mapping involves children independently choosing the referent of novel words, in contrast to children passively hearing others explicitly name novel objects. Memory for novel words could be enhanced by coupling the self-driven experience of determining the referents of novel words with children’s sense that some objects are associated with them and other objects with another character. Three-year-old children were included in the current study as Ross et al. (2011) found that 3-year-old children demonstrated an SRE and Mood (1979) found that preschool children were better at comprehending sentences that involved themselves compared to others. Children as young as two demonstrate an understanding of ownership and can distinguish between objects belonging to them and another (Eisenberg-Berg et al., 1981; Ross, 1996; Fasig, 2000). Ross (1996) found that 2-year-old children argue about ownership rights in disputes about toys. In puppet show displays, 2-year-old children are sensitive to aspects of ownership (Friedman and Neary, 2008; Vaish et al., 2011; Kanngiesser and Hood, 2014).

There is also an initial visual bias in ownership manipulations, but with development children have a good understanding of ownership with verbal information alone (Blake and Harris, 2011). Blake et al. (2012) found that 2.5-year-old children understood ownership manipulations when previously seen toys were absent while being told which objects belonged to whom suggesting that verbal information can be sufficient at this age.

It was expected that if the SRE is also applicable to child word learning then children would have enhanced memory for words associated with themselves as compared to another. After each fast mapping trial, children saw half of the novel objects enter a box assigned as theirs while being told that the objects belonged to them (self-reference); and the other objects entered another box while being told they belonged to a teddy bear (other-reference). Retention was tested immediately after fast mapping, approximately 4 h later, and the following morning to test for retention across time. Previous studies have shown that without any memory supports, 2-year-old children demonstrate poor retention of four fast-mapped words (Axelsson and Horst, 2013), but Horst and Samuelson (2008) found above-chance retention of four novel words when 2-year-old children were provided with ostensive naming following fast mapping. Vlach and Sandhofer (2012) found that when provided with memory supports, such as target label repetition or increased target salience, 3-year-old children can remember fast-mapped words up to 1 week later. Furthermore, toddlers as young as 15 months demonstrate familiarity with word sequences after a 4-h delay (Gómez et al., 2006). Williams and Horst (2014) found that 3-year-old children can remember two novel words heard during storybook reading after a 2.5-h break and 24-h later. Therefore, it was expected that 3-year-old children could retain up to four novel words in the current study immediately after fast mapping, 4 h later, and potentially the following morning.

To further explore the role of the referencing manipulation, children’s looking times during the self-/other-reference presentations were recorded to determine whether the degree of attention during the referencing presentations was associated with retention (Bergelson and Swingley, 2013; Bion et al., 2013). Bion et al. (2013) found that the length of time 2.5-month-old children looked at presentations of word-object associations was associated with longer looking at the correct targets during retention trials while other studies have not found the same association (e.g., Booth et al., 2008; Smith and Yu, 2013). Of question was whether longer looking, which is an indirect measure of attention (Holmqvist et al., 2011), would be associated with a greater degree of comprehension of the self-reference manipulation, and in turn better retention of self- and/or other-referenced words.

## Materials and Methods

### Participants

There were 23 monolingual 3-year-old children in the final sample (10 female, *M* age = 37 months 13 days, *SD* = 2 months 7 days, range = 33 months 29 days – 40 months 24 days). One participant refused to wear the required sticker for remote eye-tracking, and only accuracy data was obtained. Two further participants were tested, but due to eye-tracking difficulties causing excessive delays between fast mapping and referencing trials, their data was excluded. Participants were recruited via childcare centers and social media. Ethical approval was obtained by the university Human Research Ethics Committee.

### Stimuli and Materials

#### Nouns and Objects

**FIGURE 1:**
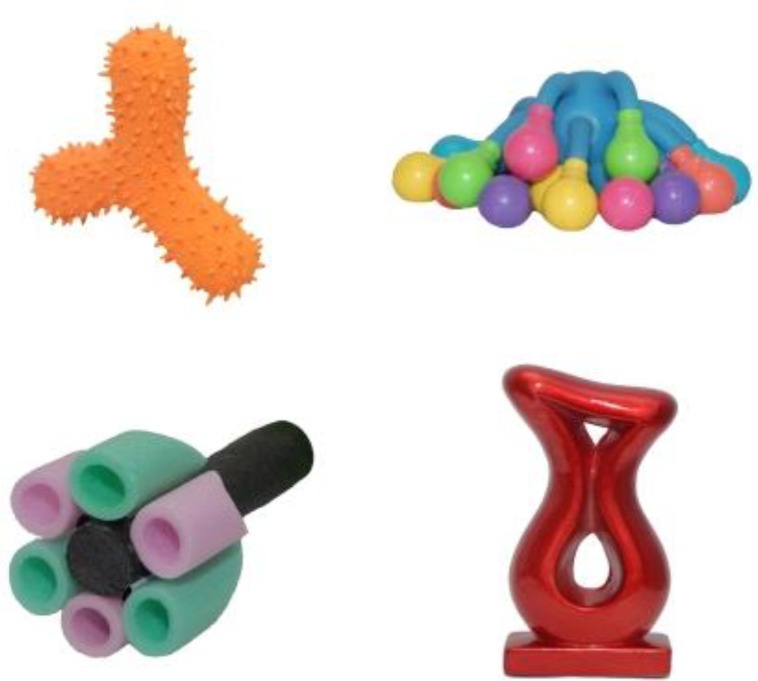
Novel targets.

Familiar nouns were selected from the OZI, an Australian version (Kalashnikova et al., 2016) of the MacArthur-Bates Cognitive Development Inventory (Fenson et al., 1993). Four novel objects and labels (see **Figure 1**) were selected from the Novel Object and Unusual Name Database (Horst and Hout, 2016). Four words were deemed sufficiently challenging for 3-year-old children (see Axelsson and Horst, 2013). The average object sizes were 53 mm × 79 mm (4.66° × 6.96° at a 65 cm distance).

#### Referencing Familiarization

**FIGURE 2:**
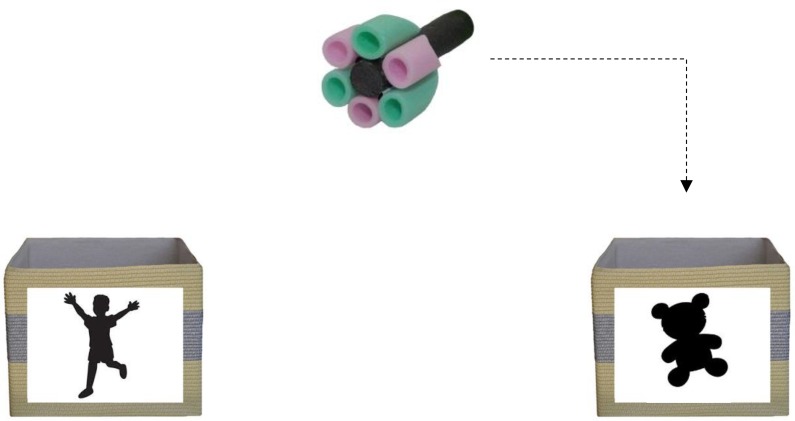
Child/self- and teddy/other-reference boxes.

To initiate children with the self-reference manipulation, children were presented with two boxes that would later be displayed on the computer during the eye-tracking session. One box had an image of a child silhouette (self-reference) and the other a teddy (other-reference) (see **Figure 2**). Children were given several toys that they could choose to insert into the child’s and/or teddy’s boxes. As in Ross et al. (2011), before proceeding, children were also asked specifically to put some toys into “their” box or in the teddy’s box to ensure that that they could comprehend the ownership aspect of the task and distinguish the boxes.

#### Eye-Tracking and Retention

Participants’ fixations were recorded with an EyeLink 1000 (SR Research^1^) eye-tracker. With the ‘Remote’ setting, allowing movement in a space of 22 cm × 18 cm × 20 cm, the sampling rate is 500 Hz with a 0.5° spatial accuracy. A high contrast sticker was placed on each toddler’s forehead above the right eye. Children sat on a booster seat 55–60 cm from the camera and 60–65 cm from the display screen. The camera was positioned in front of and beneath a 24-inch Dell monitor and the images were presented in an area with a resolution of 1024 pixels × 768 pixels (40 cm × 30 cm, 34.21° × 25.99° at a 65 cm distance). Experiment Builder software (1.10.1630) was used to present the experiment and the animated calibration points. Retention 4 h later and the following morning was completed in each child’s home with an iPad (4th generation, programmed using Xcode 6).

### Procedure and Design

After familiarization with the physical versions of the self-/other-referencing boxes, children saw a short movie on the display screen featuring dynamic abstract shapes while the experimenter ensured the camera was at an ideal height and distance. The EyeLink host computer was used to calibrate and validate children’s fixations (to approximately <2.5°) using a dynamic attention-getter (enlarging and contracting geometric shape) appearing in five points (cross pattern). Before each trial an animated attention getter (e.g., barking dog) was presented in the center of the screen and trials commenced only after children fixated the attention getter for 300 ms.

#### Familiarization Trials

The first three trials contained only familiar objects and were aimed at ensuring children could understand the task. Objects were positioned equidistant across the left, middle, and right of the screen. Children heard three sentences, e.g., “*Can you see the target? Point to the target. Where is the target?*” (duration 6 s) on a loop until children pointed or until a limit of 30 s. The experimenter pressed the space bar once the child pointed to their selection, which ended the trial. Children were praised when accurate, and corrected when inaccurate.

#### Fast Mapping Trials

Following successful completion of training, children completed 16 fast mapping trials. Each trial contained one novel object and two familiar objects, and in each trial children were asked to point to one target. In eight of the trials, the target was a novel object, and for the remaining trials the target was a familiar object. Familiar target trials were included to ensure children were selecting targets on the basis of the words and not just choosing novel items. Novel and familiar target trials alternated and each novel object was the target twice, but never in succession. The order in which novel targets appeared first was counterbalanced. Trials continued in the same manner as during the familiarization trials, except no feedback was provided.

#### Referencing

The self-reference manipulation was implemented using an ownership paradigm (e.g., van den Bos et al., 2010). After each fast mapping trial, children saw the target object from the preceding fast mapping trial presented in the top-center of the display and heard, “*The target is yours/Teddy’s! This is your/Teddy’s target. Watch the target go into your/Teddy’s box.*” (5 s) before seeing the target object move along a 90° angled path to the left or right of the screen and down into either the child’s or Teddy’s box situated on the corresponding bottom left or right of the screen (2 s) (see **Figure 2**). The size of the boxes on the display was 70 mm × 120 mm (6.16 × 10.55° at a 65 cm distance). Half of the novel objects were presented as belonging to the child and half the teddy. Whether the items were self- or other-referenced, and which side the target box appeared, was counterbalanced across four different versions of the experiment. Using SR Research Data Viewer software, interest areas (IAs) were added to the target object as the audio announced whether the object was assigned to them or to the teddy. Dynamic IAs were applied to the movement along the 90° angled path of the target from the top center of the screen to either the child’s or teddy’s box. Total looking times (dwell time, DT) during the audio and movement to the relevant box was summed and averaged across self- and other-referenced trials.

#### Retention Phases

There were three retention phases: immediately after fast mapping at the lab, roughly 4 h later (afternoon), and the following morning (overnight). Only the four novel objects were presented during the retention trials and each object appeared in one of four quadrants of the screen (see **Figure 1**). During immediate retention, there was one familiar target trial with four familiar objects to familiarize children with the new layout. This was followed by four novel target retention trials and the target location differed across trials. Each novel object was a target once. Novel target trials were presented in the same order as they appeared across the fast mapping trials. The same procedure as during the fast mapping trials were followed and children were asked to point to a target in each trial. The two delayed retention tests were completed with an iPad and children were asked to touch the target image on the screen. There were four familiarization trials with only familiar items before each delayed test to ensure children were engaged with the task prior to completing the four novel target retention trials. In all three retention phases, feedback was provided during the familiarization trials, but not the novel target trials. The fast mapping and immediate retention trials were video-taped and coded offline by a second blinded experimenter for 20% (*n* = 5) of the participants. Inter-coder reliability was high (*r* = 0.92).

## Results

SPSS Version 22 was used to analyze the data. As most of the variables were negatively skewed, non-parametric analyses were performed. Mann–Whitney *U* tests were used to compare retention scores to chance and Wilcoxon Signed Rank tests for the paired comparisons. Correlations were analyzed using Spearman’s Rank-Order tests.

### Familiarization

**FIGURE 3:**
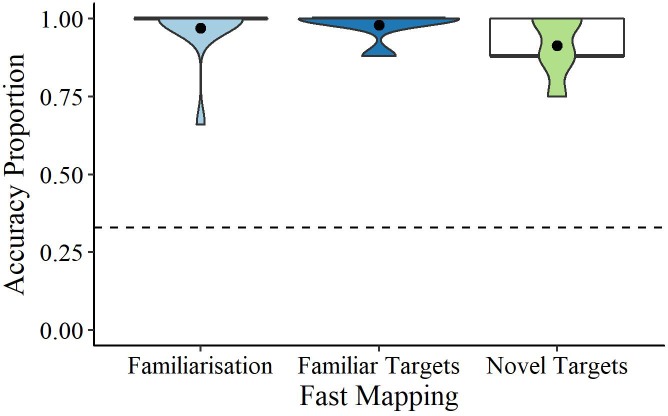
Violin and boxplots of accuracy scores for familiarization, and familiar and novel target fast mapping trails. Dots denote the means, bold lines the medians, and the dashed line chance (0.33).

These trials contained only familiar objects and children’s median accuracy was compared to chance (0.33 due to a choice of three items). The median accuracy was significantly higher than chance (0.33), *U* = 276.00, *p* < 0.001, *r* = 0.96, indicating that children could successfully point to the targets in response to the audio (see **Figure 3**).

### Fast Mapping

For the familiar target trials, children’s selections were significantly higher than chance (0.33 due to a choice of three items, one novel, two familiar), *U* = 276.00, *p* < 0.001, *r* = 0.94. Children’s accuracy in the novel target trials was also significantly higher than chance (0.33), *U* = 276.00, *p* < 0.001, *r* = 0.89 (see **Figure 3**). There was no significant difference in fast mapping accuracy for novel target items that were subsequently self- or other-referenced, *Z* = 1.16, *p* = 0.248, *r* = 0.23.

### Retention

#### Accuracy

**FIGURE 4:**
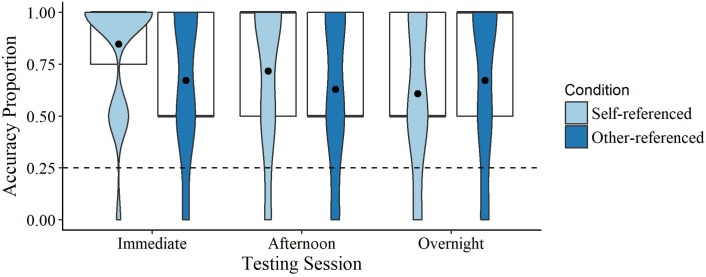
Violin and boxplots of retention accuracy scores for self- and other-referenced items. Dots denote the means, bold lines the medians, and the dashed line chance (0.25).

The difference in dwell time (DT) during the self-reference (*Mdn* = 2594, *M* = 2384 ms, *SD* = 757) and other-reference trials (*Mdn* = 2428, *M* = 2515 ms, *SD* = 795) was non-significant, *Z* = 0.83, *p* = 0.408, *r* = 0.18, indicating that the time spent looking at the target during both the self- and other-referencing trials was similar. Children’s immediate retention for both the self- (*U* = 272.50, *p* = <0.001, *r* = 0.89) and other-referenced novel targets (*U* = 256.50, *p* = <0.001, *r* = 0.77) were significantly higher than chance (0.25 due to the presence of four items). Immediate retention for the self-referenced items was also significantly higher than other-referenced items, *Z* = 2.14, *p* = 0.033, *r* = 0.49 (see **Figure 4**). Delayed afternoon retention was also higher than chance for both self- (*U* = 259.50, *p* = <0.001, *r* = 0.79) and other-referenced targets (*U* = 248.00, *p* = <0.001, *r* = 0.72), but the difference between the two was no longer significant, *Z* = 0.88, *p* = 0.378, *r* = 0.18. Overnight retention was also higher than chance for self- (*U* = 246.00, *p* = <0.001, *r* = 0.71) and other-referenced targets (*U* = 252.00, *p* = <0.001, *r* = 0.74), but the difference between the two conditions was non-significant, *Z* = 0.78, *p* = 0.464, *r* = 0.16 (see **Figure 4**).

#### FM Accuracy and Retention Accuracy

There was a significant positive relationship between children’s fast mapping of self-referenced targets and immediate retention for self-referenced items, *r*_s_(22) = 0.47, *p* = 0.022. The relationship between fast mapping accuracy for other-referenced items and immediate retention was non-significant, *r*_s_(22) = -0.23, *p* = 0.302. All relationships between fast mapping and delayed afternoon retention and overnight retention were non-significant.

#### Reference Trials Dwell Time (DT) and Accuracy

Correlations between the total looking time during the self-/other-reference trials and retention accuracy for the corresponding words was assessed using Bonferroni corrections for each retention phase (0.05/3 = 0.0167). Only DT during the self-reference trials was positively associated with afternoon accuracy for self-referenced items. None of the other correlations were significant (see **Table 1**), but it suggests that the longer children looked during the self-reference trials, the better their afternoon retention for self-referenced words.

**Table 1:** Relationship (Spearman’s *r*) between mean dwell time (DT) during self- and other-reference trials and retention accuracy for corresponding words.

		Immediate	Afternoon	Overnight
		retention	retention	retention
Reference		
Self	*r_s_*	0.21	0.53^∗^	0.02
	*(p)*	(0.340)	(0.010)	(0.918)
Other	*r_s_*	0.33	0.24	0.47
	*(p)*	(0.136)	(0.289)	(0.029)

^∗^*Bonferroni corrections applied (0.05/3 = 0.0167)*.

#### Reference Trial Dwell Time (DT) and Immediate Retention Proportional DT

Proportional looking to each target in each retention trial was calculated by dividing the total looking time to each target by the sum of the total looking times to all objects. The mean proportional looking to the self- and other-referenced objects were then calculated. The relationship between DT in the self- and other-reference trials and mean proportional DT to the corresponding targets in the immediate retention phase was assessed. Note, the difference in proportional DT to self- (*Mdn* = 0.20, *M* = 0.25, *SD* = 0.22) and other-referenced items (*Mdn* = 0.14, *M* = 0.16, *SD* = 0.14) during the retention trials was non-significant, *Z* = 0.98, *p* = 0.324, *r* = 0.23. The retention period was defined as starting from 367 ms post target noun onset (see Swingley, 2009) to the point at which children pointed to their chosen object (based on the experimenter space bar press; see section “Procedure”). The relationship between DT during self-reference trials and proportional DT to self-referenced targets during retention was non-significant, *r*(20) = 0.002, *p* = 0.994, but there was a significant, positive relationship between DT during other-reference trials and proportional DT to other-referenced items in the retention trials *r*(20) = 0.62, *p* = 0.003. Longer looking during other-reference trials was associated with a greater proportion of time spent looking at other-referenced targets during retention relative to the competitors at test.

## Discussion

A test of the SRE on children’s retention of fast-mapped words demonstrated that 3-year-old children retained novel words at levels better than chance for items presented as belonging to them or another character after each fast mapping trial. Notably, retention of self- compared to other-referenced items was significantly higher at the immediate retention test. Four hours later and the following morning, children’s retention of self- and other-referenced words were still significantly above chance, but at similar levels. In adults, self- compared to other-reference effects are smaller than self- compared to semantically encoded effects (see Symons and Johnson, 1997). It appears that in relation to fast mapping in children, self-referencing provides an immediate advantage for remembering self- compared to other-referenced words, but the difference diminishes over time. It is also likely that both forms of referencing are an effective form of explicit renaming or ostensive naming (e.g., Horst and Samuelson, 2008), but it is likely that self-reference provides a greater initial facilitative effect on memory.

Fast mapping of self-related items was positively associated with immediate retention for self-related items and longer looking during the self-reference manipulation was associated with better retention for self-referenced objects 4 h later. The looking times during the other-reference trials were also associated with greater proportional looking at the other-referenced items during the immediate retention trials, which suggests that longer looking during the other-reference trials was associated with more focussed looking at other-referenced targets relative to the competitors during retention. Bion et al. (2013) also found that 2.5-year-old children’s looking times during presentations of word-object associations was associated with longer looking at the targets during retention, but others have not found the same relationship (e.g., Booth et al., 2008; Smith and Yu, 2013). In the current study, children were also required to point to the target. It is difficult to determine what greater proportional looking to other-referenced items during retention indicates. Children’s greater degree of attention, while being shown which items ‘belonged’ to Teddy, might have helped to highlight those word-object associations during retention. However, greater proportional looking to other-referenced items during retention could also indicate that children were less certain about those items and looked more at them before pointing. As the correlation between looking times during the other-reference trials was only significantly related to proportional *looking* at other-referenced targets during retention, but not retention *accuracy* for other-referenced targets, the effect of other-referencing was perhaps more implicit in nature. These findings raise further questions about the role of attention during encoding and later retention.

Much like the findings with adults (e.g., Cunningham et al., 2008; van den Bos et al., 2010) and children (Sui and Zhu, 2005; Cunningham et al., 2013), telling participants that objects belonged to them as opposed to another character was followed by enhanced retention in the immediate test. Further, 3-year-olds are the youngest reported age group to demonstrate an SRE (Ross et al., 2011), and the results here provide further support that children at this age not only comprehend the ownership manipulation, but might also experience memory-related benefits of a sense of ownership^2^. Encoding novel words in relation to oneself could therefore enhance retention as knowledge of oneself is richer than other topics and this is argued to facilitate associative links and organization of new information in current memory stores, and in turn enhance retrieval pathways (e.g., Klein, 2012). According to van den Bos et al. (2010), memory for one’s own items might be enhanced because issues surrounding ownership might enhance attention and emotional arousal particularly to self-referenced items. The looking times during the self- and other-referencing trials were similar, but total looking time is only an indirect measure of attention (Holmqvist et al., 2011). Children as young as two demonstrate emotional responses to ownership issues (e.g., Ross, 1996), but as argued by Klein (2012) there might have been a combination of greater elaboration and organization of self-referenced novel words in memory and stronger emotional arousal during self-referencing (van den Bos et al., 2010) that could explain the immediate retention results. Future studies could incorporate physiological measures to see if there is an association with better memory for self-referenced words.

What is less clear is whether children’s above-chance retention was due to the ostensive naming, repetition, and highlighting effect that the self-reference presentations provided rather than the ownership manipulation itself (e.g., Horst and Samuelson, 2008; Gurteen et al., 2011; Axelsson et al., 2012; Vlach and Sandhofer, 2012). This is particularly the case for the afternoon and overnight tests, as retention was significantly greater than chance, but there was no longer better retention for self-referenced words. Ross et al. (2011) found an SRE with 3-year-old, but not 4-year-old children and Sui and Zhu (2005) found an SRE with 5-year-old, but not 10-year-old children, but after increasing task difficulty by adding more items to remember all age groups demonstrated an SRE. In adults, shorter presentation times during encoding are associated with a stronger SRE (Symons and Johnson, 1997). Therefore, if the task is too easy an SRE is less likely as items assigned to both the self and other are both easily retained. Increasing the number of words and objects could help determine if the items associated with oneself is stronger and sustainable over time.

The SRE is associated with greater specific as opposed to ambiguous memories for self-referential items in adults, which could suggest an episodic aspect to the memory of self-referential items (Conway et al., 2001; van den Bos et al., 2010). However, it is possible that children in the current study had forgotten which items belonged to whom, but retained the word-object associations and this could explain why the SRE diminished. Ross et al. (2011) argued that young children might not retain explicit memories of referencing, and that their memories of ownership manipulations might be more implicit in nature leading to a bias for self-referent items. This could explain why the effect was not sustained over time. However, Ross et al. (2011) further argued that a delayed effect might not be necessary as it is the early association between the self and the stimuli during encoding that is key to the effect.

In previous studies of the SRE in children (Sui and Zhu, 2005; Ross et al., 2011; Cunningham et al., 2013), the stimuli were familiar objects. In the current study, the task involved retention of novel word-object associations. Therefore, the task was arguably more challenging, requiring not only forming an association between words and objects, but also creating links with either themselves or another character. Word learning involves a slow and gradual strengthening of association between words and objects over time and without further exposure to the correct word-object links, the association weakens (e.g., McMurray et al., 2012). It is likely therefore that the association with the ownership manipulation is also weakened. The SRE ownership manipulation is argued to involve ‘self-conservation’ where a current representation of oneself is linked with a previous representation of oneself during encoding (Fasig, 2000). It is possible that this link weakened over time. Children could be asked if they remember which items were assigned to them or the alternative character to determine whether they explicitly remembered the self-/other-reference manipulation and whether that is related to the presence or absence of an SRE.

There was greater retention for self- compared to other-related items in the immediate test and a positive relationship between looking times during self-related ownership trials and afternoon retention accuracy. This suggests that the self-related ownership manipulation might have had a relevant memory enhancing effect, but this should be interpreted with caution and comparisons to other conditions are also required to determine if self-referencing can explain the enhanced immediate retention. Future studies should compare self- and other-referencing with simple ostensive naming of the novel words or to no form of memory support to determine the degree of the effect of the SRE manipulation. A referencing condition could be added where the objects are inserted into boxes, but in the absence of an ownership manipulation. Kucker and Samuelson (2012) found that greater visual exposure to objects can enhance delayed retention of associated words. Therefore, further exposure to the objects entering boxes alone could have an effect on retention.

Children in the current study played a rather passive role in that they were told whether an object belonged to them or the teddy. Of question is how much children understood the ownership manipulation by listening to audio and watching objects move into boxes. In previous studies, children played a more active role such as placing images of objects belonging to them or another child into separate boxes (Cunningham et al., 2013), or talking about pictures of themselves performing actions with objects (Ross et al., 2011). Cunningham et al. (2014) found an SRE in a study where 4- to 6-year-olds were asked whether they or another child would like a series of objects using images presented on a computer screen. This was not an ownership-related SRE paradigm, but does suggest that the children could get a sense of an association between themselves and a number of objects separate to another character presented on a screen. Children as young as two demonstrate comprehension from video screens (Pempek et al., 2010), and can also make use of information presented to them on screens (Troseth, 2003), particularly when there is interaction involved (Troseth et al., 2006; Kirkorian and Choi, 2017). Krcmar and Cingel (2017) found that toddlers learned novel words via video better if the speaker spoke directly to the child and encouraged a response as opposed to when watching from a third person perspective. In the current study, children pointed in response to the instructions communicated directly to the them and the referencing trials also involved direct communication with the child. Fast mapping involves independent determination of the association between words and referents, but future investigations could test the effects of self-reference on retention of fast-mapped words where children are involved in the self-/other-reference manipulation such that they could be asked to move the objects into the boxes themselves using a touch screen.

The children in the current study were told prior to the experiment that the silhouettes on the boxes represented them and the teddy, and the experimenter checked that they could distinguish between the silhouettes that represented them and the teddy. However, images of the children themselves, as used by Ross et al. (2011), would likely have a stronger effect as the children are likely to form a stronger association between their box and themselves. Nonetheless, the findings here suggest that providing further associative word-object presentations in relation to ownership has immediate benefits for those related to oneself. An ownership manipulation could be a beneficial form of memory support for children’s retention of novel words, but further testing is required to determine whether the findings are due to the self- and other-referencing or whether it is due to the accompanying repetition and re-exposure to the words that has an enhancing effect on children’s retention of novel words.

## Ethics Statement

This study was carried out in accordance with the recommendations of National Statement on Ethical Conduct in Human Research, Australian Research Council and National Health and Medical Research Council. The protocol was approved by the Australian National University Ethics Committee. All parents of the children gave written informed consent in accordance with the Declaration of Helsinki.

## Author Contributions

EA and RD wrote the manuscript. EA, RD, SY, and TQ were involved in study design, data collection, analyses, and contributed intellectually to the manuscript.

## Conflict of Interest Statement

The authors declare that the research was conducted in the absence of any commercial or financial relationships that could be construed as a potential conflict of interest.

## Footnotes

1 http://www.sr-research.com

2 Children often expressed excitement at being told that objects belonged to them during the eye-tracking task.

## References

[B1] Axelsson E. L. Churchley K. Horst J. S. (2012). The right thing at the right time: why ostensive naming facilitates word learning. *Front. Psychol.* 3:88. 10.3389/fpsyg.2012.00088 22470363 PMC3314248

[B2] Axelsson E. L. Horst J. S. (2013). Testing a word is not a test of word learning. *Acta Psychol.* 144 264–268. 10.1016/j.actpsy.2013.07.002 23928497

[B3] Bergelson E. Swingley D. (2013). Young toddlers’ word comprehension is flexible and efficient. *PLoS One* 8:e73359. 10.1371/journal.pone.0073359 23991189 PMC3749997

[B4] Bion R. A. Borovsky A. Fernald A. (2013). Fast mapping, slow learning: disambiguation of novel word–object mappings in relation to vocabulary learning at 18, 24, and 30 months. *Cognition* 126 39–53. 10.1016/j.cognition.2012.08.008 23063233 PMC6590692

[B5] Blake P. Harris P. (2011). Early representations of ownership. *New Dir. Child Adolesc. Dev.* 2011 39–51. 10.1002/cd.295 21671340

[B6] Blake P. R. Ganea P. A. Harris P. L. (2012). Possession is not always the law: with age, preschoolers increasingly use verbal information to identify who owns what. *J. Exp. Child Psychol.* 113 259–272. 10.1016/j.jecp.2012.06.008 22832198

[B7] Booth A. E. McGregor K. K. Rohlfing K. J. (2008). Socio-pragmatics and attention: contributions to gesturally guided word learning in toddlers. *Lang. Learn. Dev.* 4 179–202. 10.1080/15475440802143091

[B8] Carey S. (1978). “The child as a word learner,” in *Linguistic Theory and Psychological Reality*, eds Halle M. Bresnan J. Miller G. A. (Cambridge, MA: MIT Press).

[B9] Conway M. A. Dewhurst S. A. Pearson N. Sapute A. (2001). The self and recollection reconsidered: how a “failure to replicate” failed and why trace strength accounts of recollection are untenable. *Appl. Cogn. Psychol.* 15 673–686. 10.1002/acp.740

[B10] Cunningham S. J. Brebner J. L. Quinn F. Turk D. J. (2014). The self-reference effect on memory in early childhood. *Child Dev.* 85 808–823. 10.1111/cdev.12144 23888928

[B11] Cunningham S. J. Turk D. J. MacDonald L. M. Macrae C. N. (2008). Yours or mine? Ownership and memory. *Conscious. Cogn.* 17 312–318. 10.1016/j.concog.2007.04.003 17543540

[B12] Cunningham S. J. Vergunst F. Macrae C. N. Turk D. J. (2013). Exploring early self-referential memory effects through ownership. *Br. J. Dev. Psychol.* 31 289–301. 10.1111/bjdp.12005 23901843

[B13] Eisenberg-Berg N. Haake R. J. Bartlett K. (1981). The effects of possession and ownership on the sharing and proprietary behaviors of preschool children. *Merrill Palmer Q.* 27 61–68.

[B14] Fasig L. G. (2000). Toddlers’ understanding of ownership: implications for self-concept development. *Soc. Dev.* 9 370–382. 10.1111/1467-9507.00131

[B15] Fenson L. Dale P. S. Reznick J. S. Thal D. Bates E. Hartung J. P. (1993). *The MacArthur Communicative Development Inventories: User’s Guide and Technical Manual*. San Diego, CA: Singular Publishing Group.

[B16] Friedman O. Neary K. R. (2008). Determining who owns what: do children infer ownership from first possession? *Cognition* 107 829–849. 10.1016/j.cognition.2007.12.002 18243169

[B17] Gómez R. L. Bootzin R. R. Nadel L. (2006). Naps promote abstraction in language-learning infants. *Psychol. Sci.* 17 670–674. 10.1111/j.1467-9280.2006.01764.x 16913948

[B18] Gurteen P. M. Horne P. J. Erjavac M. (2011). Rapid word learning in 13- and 17-month-olds in a naturalistic two-word procedure: looking versus reaching measures. *J. Exp. Child Psychol.* 109 201–217. 10.1016/j.jecp.2010.12.001 21216414

[B19] Halberda J. (2003). The development of a word-learning strategy. *Cognition* 87 B23–B34. 10.1016/s0010-0277(02)00186-5 12499109

[B20] Holmqvist K. Nyström M. Andersson R. Dewhurst R. Halszka J. van de Weijer J. (2011). *Eye-Tracking: A Comprehensive Guide to Methods and Measures*. Oxford: Oxford University Press.

[B21] Horst J. S. Hout M. C. (2016). The novel object and unusual name (NOUN) database: a collection of novel images for use in experimental research. *Behav. Res. Methods* 48 1393–1409. 10.3758/s13428-015-0647-3 26424438

[B22] Horst J. S. Samuelson L. K. (2008). Fast mapping but poor retention by 24-month-old infants. *Infancy* 13 128–157. 10.1080/15250000701795598 33412722

[B23] Kalashnikova M. Schwarz I.-C. Burnham D. (2016). OZI: Australian english communicative development inventory. *First Lang.* 36 407–427. 10.1177/0142723716648846

[B24] Kanngiesser P. Hood B. M. (2014). Young children’s understanding of ownership rights for newly made objects. *Cogn. Dev.* 29 30–40. 10.1016/j.cogdev.2013.09.003

[B25] Kirkorian H. L. Choi K. (2017). Associations between toddlers’ naturalistic media experience and observed learning from screens. *Infancy* 22 271–277. 10.1111/infa.12171 33158337

[B26] Klein S. B. (2012). Self, memory, and the self-reference effect an examination of conceptual and methodological issues. *Pers. Soc. Psychol. Rev.* 16 283–300. 10.1177/1088868311434214 22291045

[B27] Klein S. B. Loftus E. M. (1988). The nature of self-referent encoding: the contribution of elaborative and organisational processes. *J. Pers. Soc. Psychol.* 55 5–11. 10.1037/0022-3514.55.1.5

[B28] Krcmar M. Cingel D. P. (2017). Do young children really learn best from the use of direct address in children’s television? *Media Psychol.* 1 1–20. 10.1080/15213269.2017.1361841

[B29] Kucker S. C. Samuelson L. K. (2012). The first slow step: differential effects of object and word-form familiarization on retention of fast-mapped words. *Infancy* 17 295–323. 10.1111/j.1532-7078.2011.00081.x 22661907 PMC3362040

[B30] McMurray B. Horst J. S. Samuelson L. K. (2012). Supplemental material for word learning emerges from the interaction of online referent selection and slow associative learning. *Psychol. Rev.* 119 831–877. 10.1037/a0029872.supp 23088341 PMC3632668

[B31] Mervis C. B. Bertrand J. (1994). Acquisition of the Novel Name-Nameless Category (N3C) principle. *Child Dev.* 65 1646–1662. 10.2307/1131285 7859547

[B32] Mood D. W. (1979). Sentence comprehension in preschool children: testing an adaptive egocentrism hypothesis. *Child Dev.* 50 247–250. 10.2307/1129064 446210

[B33] Munro N. Baker E. McGregor K. Docking K. Arculi J. (2012). Why word learning is not fast. *Front. Psychol.* 3:41. 10.3389/fpsyg.2012.00041 22393326 PMC3289981

[B34] Pempek T. A. Kirkorian H. L. Richards J. E. Anderson D. R. Lund A. F. Stevens M. (2010). Video comprehensibility and attention in very young children. *Dev. Psychol.* 46 1283–1293. 10.1037/a0020614 20822238 PMC2936722

[B35] Rogers T. B. Kuiper N. A. Kirker W. S. (1977). Self-reference and the encoding of personal information. *J. Pers. Soc. Psychol.* 35 677–688. 10.1037/0022-3514.35.9.677 909043

[B36] Ross H. S. (1996). Negotiating principles of entitlement in sibling property disputes. *Dev. Psychol.* 32 90–101. 10.1037/0012-1649.32.1.90

[B37] Ross J. Anderson J. R. Campbell R. N. (2011). I remember me: mnemonic self-reference effects in preschool children. *Monogr. Soc. Res. Child Dev.* 76 1–102. 10.1111/j.1540-5834.2011.00614.x

[B38] Smith L. B. Yu C. (2013). Visual attention is not enough: individual differences in statistical word-referent learning in infants. *Lang. Learn. Dev.* 9 37–41. 10.1080/15475441.2012.707104 24403867 PMC3882028

[B39] Sui J. Zhu Y. (2005). Five-year-olds can show the self-reference advantage. *Int. J. Behav. Dev.* 29 382–387. 10.1080/01650250500172673

[B40] Swingley D. (2009). Contributions of infant word learning to language development. *Philos. Trans. R. Soc. Lond. B Biol. Sci.* 364 3617–3632. 10.1098/rstb.2009.0107 19933136 PMC2828984

[B41] Symons C. S. Johnson B. T. (1997). The self-reference effect in memory: a meta-analysis. *Psychol. Bull.* 121 371–394. 10.1037/0033-2909.121.3.371 9136641

[B42] Troseth G. L. (2003). TV guide: two-year-old children learn to use video as a source of information. *Dev. Psychol.* 39 140–150. 10.1037/0012-1649.39.1.140 12518815

[B43] Troseth G. L. Saylor M. M. Archer A. H. (2006). Young children’s use of video as a source of socially relevant information. *Child Dev.* 77 789–799. 10.1111/j.1467-8624.2006.00903.x 16686801

[B44] Vaish A. Missana M. Tomasello M. (2011). Three year old children intervene in third party moral transgressions. *Br. J. Dev. Psychol.* 29 124–130. 10.1348/026151010X532888 21288257

[B45] van den Bos M. Cunningham S. J. Turk D. J. (2010). Mine to remember: the effects of minimal ownership on remembering and knowing. *Q. J. Exp. Psychol.* 63 1065–1071. 10.1080/17470211003770938 20401814

[B46] Vlach H. A. Sandhofer C. M. (2012). Fast mapping across time: memory processes support children’s retention of learned words. *Front. Psychol.* 3:46. 10.3389/fpsyg.2012.00046 22375132 PMC3286766

[B47] Williams S. E. Horst J. S. (2014). Goodnight book: sleep consolidation improves word learning via storybooks. *Front. Psychol.* 5:184. 10.3389/fpsyg.2014.00184 24624111 PMC3941071

